# Endometrial large cell neuroendocrine carcinoma: A case report and literature review

**DOI:** 10.1016/j.gore.2024.101429

**Published:** 2024-06-03

**Authors:** Feng Yang, Shoujun Liang, Chuanzhong Liu, Yeping Wei, Liying Zhang

**Affiliations:** Gynecology Department, The Second Affiliated Hospital of Guangxi Medical University, Nanning 530007, China

**Keywords:** Large cell neuroendocrine carcinoma, Endometrium, Immunohistochemistry

## Abstract

Endometrial large cell neuroendocrine carcinoma (LCNEC) is a highly malignant tumor that presents with neuroendocrine function. It is difficult to diagnose at an early stage. Moreover, the diagnosis depends on the pathological and immunohistochemical findings. It is also prone to distant metastasis, but is difficult to treat and shows poor prognosis. Presently, there exists no unified treatment plan, and the prognosis of this disease is also poor. We reported here an analysis and literature review of a case of endometrial LCNEC to facilitate the comprehension of this disease and provide help toward clinical diagnosis and treatment.

## Highlights

• **Patient Presentation**: 48-year-old woman with irregular vaginal bleeding, presenting a polypoid mass protruding from cervical canal.
• **Diagnostic Findings**: Ultrasound and CT scan revealed uterine mass with necrosis, and immunohistochemistry showed characteristics of LCNEC.
• **Treatment and Outcome**: Underwent laparoscopic hysterectomy, radiotherapy, and cisplatin chemotherapy, but died of tumor recurrence.
• **Pathological Examination**: Revealed tumor infiltration into the muscular layer, with metastases observed in the ovary and abdominal lymph nodes.
• **Prognosis**: Diagnosed with stage IIIc endometrial LCNEC, ultimately leading to fatal outcome despite treatment efforts.

## 1 Introduction

Endometrial large cell neuroendocrine carcinoma (LCNEC) is a highly malignant tumor, and due to its low incidence, the clinical understanding of the biological behavior, treatment, and prognosis is poor, warranting accumulation of more clinical experience. We have analyzed and reported a case of endometrial LCNEC and reviewed the relevant literature to improve the comprehension of this disease and facilitate the development of precise clinical diagnosis and treatment.

## 2 Case summary

**Fig. 1:**
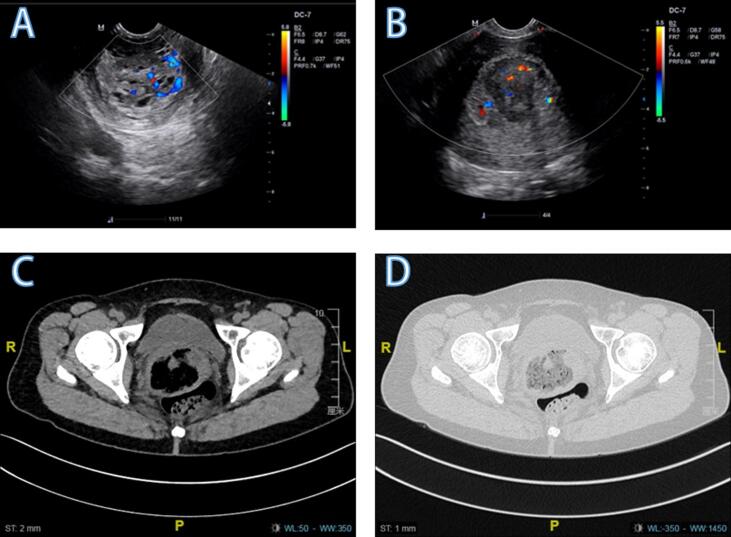
Pelvic ultrasound (A and B) and CT (C and D) showing a soft tissue mass protruding from the uterine cavity to the cervical area.

The case patient was a 48-year-old woman who presented with reports of irregular vaginal bleeding for more than 1 month. Her pelvic ultrasound examination showed the mixed echo group from the uterine cavity to the cervical canal of 9.1 × 4.9 × 3.7 cm^3^ (Fig. 1A and B). Her gynecological examination revealed a dark-red mass protruding from the cervical canal to the vagina of an approximate size 5 × 5 × 4 cm^3^, irregular in shape, with erosion and necrosis on the surface, easy to bleed on touch. The pathological examination revealed the presence of fibrous tissues, necrotic cells, and a small number of atypical cells. Immunohistochemical examination revealed the following results: Syn (+), CgA (−), CX (epithelial-), CK7 (−), P40 (−), CK5/6 (−), P63 (−), CD10 (−), P16 (partial +), S-100 (−), HMB45 (−), MelanA (−), and LCA (−). The CT revealed that the volume of the uterus was increased, with an uneven density and soft tissue mass (7.7 × 6.8 × 5.5 cm^3^) protruding from the uterine cavity to the cervical area (Fig. 1C and D). Enhanced scanning revealed slight non-uniform enhancement. No abnormalities were identified in gynecological tumor markers.

## 3 Management

**Fig. 2:**
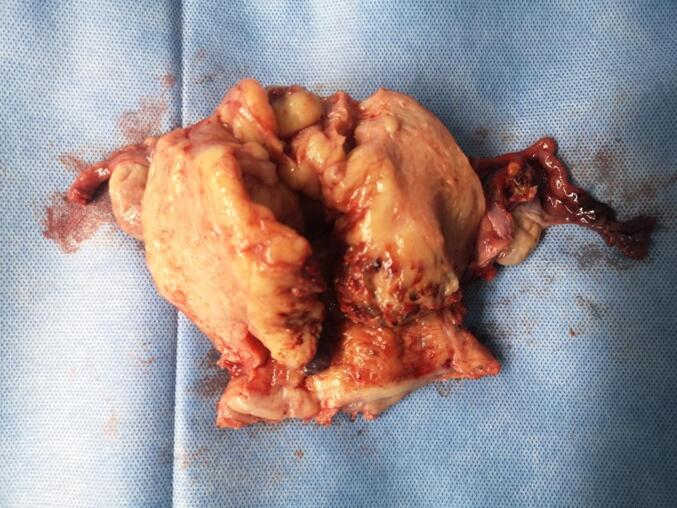
Intrauterine masses in the removd uterus.

**Fig. 3:**
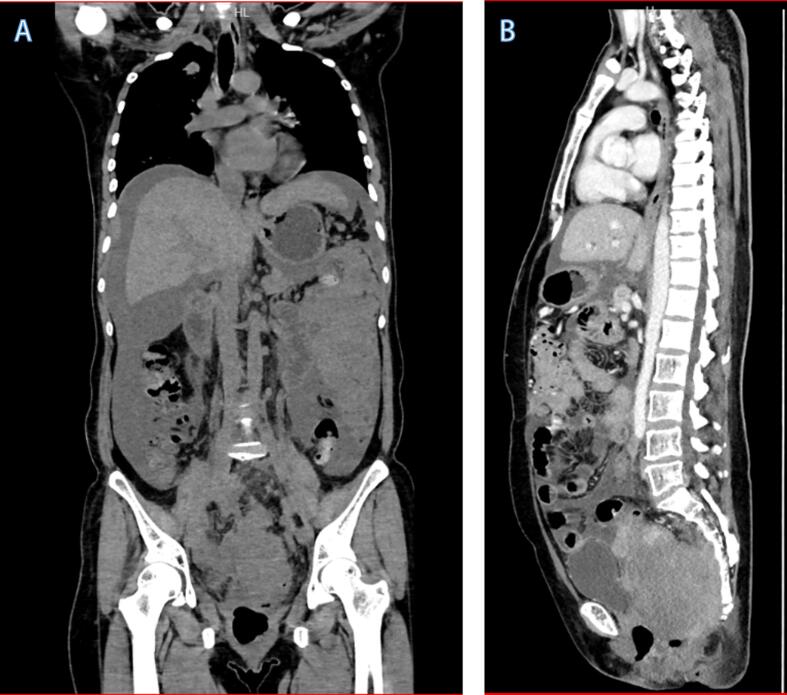
CT (A and B) showing multiorgan metastases after treatment.

On August 29, 2019, the patient received laparoscopic hysterectomy, salpingectomy, oophorectomy, pelvic lymph node dissection, and para-aortic lymph node dissection. The anatomy of the uterus revealed a polypoid mass in the uterine cavity of an approximate size 9 × 5 × 5 cm^3^ (Fig. 2). The pathological results revealed the following: malignant tumor, infiltration to the whole muscular layer, no tumor tissues in the cervix, vaginal wall incisal margin, vaginal fornix, and left and right ligaments. The tumors were observed in the right ovary and the right abdominal aortic lymph node, with a similar morphology to that of the uterine fundus. Immunohistochemistry examination revealed the following: Syn (+), Vim (less +), S100 (−), CK (−), α-inhibin (−), CD99 (−), Calretinin (−), CD10 (−), Desmin (Less +), CgA (−), CD138 (−), CD38 (−), ER (−), PR (−), WT1 (−), HMB45 (−), Ki67 (+, 40%), MyoD1 (−), and Myogenin (−), which were consistent with the results of LCNEC. With reference to the FIGO staging criteria, the patient was diagnosed with endometrial LCNEC stage IIIc. After surgery, she underwent external pelvic irradiation using conformal intensity modulated radiation therapy with dose of 200Gy/25f, and single-agent cisplatin (60mg dosage) chemotherapy concurrently with radiotherapy weekly. On November 13, the patient received chemotherapy with etoposide and cisplatin once, after which the patient declined the chemotherapy. On February 2020, she experienced abdominal distension without any obvious inducement accompanied with bloating and was re-admitted to the hospital. Her CT revealed multiple recurrences of tumors in the distal sigmoid colon, rectum, anterior sacrum, diaphragm, peritoneum, abdominal cavity, and lymph nodes (Fig. 3A and B). Considering the tumor was uncontrolled, the patient refuses to continue the treatment and started oral herbal medication, and died of the disease in March 2021.

## 4 Discussion with review of literature

### 4.1 Etiology

The diffuse neuroendocrine system (DNES) is another system that is independent of the somatic nervous system and the autonomic nervous system. It is also called the amine-precursor uptake and decarboxylation (APUD) system. Hormone-like endocrine cells and neuroendocrine carcinoma (NEC) are malignant tumors derived from APUD cells. The WHO (2020) classification of female reproductive system tumors (Höhn et al., 2021) specifies large-cell type into high-level NEC. NEC are rare in female reproductive organs, especially in the endometrium. Endometrial LCNEC is very rare with an incidence of <60 cases worldwide (Table 1).

**Table 1:** Published reports of LCNEC

Number	Date	Author	Geographical location	Age	Symptoms	Pathology	IHC staining	Tumor Size (cm)	FIGO Stage	Surgical Treatment	Adjuvant Therapy	Outcome(months)
1	2017	Aya Kobayashi	Japan	52	Abdominal pain	LCNEC	Syn,CgA,CD56	NA	IIIC	TAH,BSO,LND	RT,irinotecan,cisplatin	DOD10
2	2017	Harunobu Matusmoto	Japan	51	Uterine enlargement	LCNEC + ECa	Syn,CgA,CD56,Ki-67	3	IIIA	TAH,BSO,LND	Irinotecan, cisplatin	NED20
3	2008	Lorena Posligua	USA	59	Abnornal pap smear	LCNEC + ECa+serous component	Syn,NSE,CD56,CD57,p53,p16	12.5	IIIB	TAH,BSO,omentectomy,LND	RT,unspecfied chemo	NED92
4	2012	Natsuko Makihara	Japan	73	Abnornal distension	LCNEC	Syn,CgA,NSE,p53	NA	IVB	Refused surgery	Refused chemo	DOD1
5	2012	Natsuko Makihara	Japan	73	PMB	LCNEC	Syn,CgA,CD56,p53	NA	IIIC	TAH,BSO,omentectomy,LND	Irinotecan, cisplatin	AWD13
6	2018	Jumpei Ogura	Japan	52	AUB	LCNEC	Syn,CgA,CD56,Ki67	16.9	IIIC	none	none	DOD1
7	2007	Nicholas J.Mulvany	Australia	50	PMB	LCNEC	Syn,NSE	2.2	IIIC	TAH,BSO,omentectomy,LND	RT,carboplatin,etoposide	AWD12
8	2007	Nicholas J.Mulvany	Australia	80	PMB	LCNEC + ECa	NSE,cytokeratin,AE1/AE3	4.5	IC	TAH,BSO,LND	RT	DOD5
9	2007	Nicholas J.Mulvany	Australia	77	PMB	LCNEC + ECa	Syn,NSE,CD56	7.5	IIB	TAH,BSO	RT	DOD23
10	2007	Nicholas J.Mulvany	Australia	79	PMB	LCNEC + ECa	NSE,cytokeratin,AE1/AE3,CD56	1.5	IIIA	TAH,BSO,omental and peritoneal biopsies	RT	AWD2
11	2007	Nicholas J.Mulvany	Australia	88	PMB	LCNEC + ECa + squamous differentiation	NSE,cytokeratin,AE1/AE3,CD56	5	IIIC	TAH,BSO,LND	Paclitaxel, carboplatin	AWD1
12	2016	Kyoko Ono	Japan	41	AUB	LCNEC + ECa	Syn,CgA,CD56,CK7	10	II	TAH	Paclitaxel, carboplatin	AWD24
13	2017	Antonio Ieni	Italy	78	Colonic obstruction	LCNEC + poorly dfferentiated adenocarcinoma	Syn,CgA,CD56,Ki67,ER,PR,EMA,MLH1,MSH2,MSH6	21	IVB	Suboptimal debulking,TAH,BSO,small bowel resection,removal of sigmoid colon and portion of rectum	Unspecied chemo	AWD3
14	2018	Yi-An Tu	Taiwan	51	PMB	LCNEC	Syn,cytokeratin,p53,CD56	NA	IVB	TAH,BSO,omentectomy,Suboptimal debulking	Etoposide,cisplatin	DOD3
15	2011	Shohreh Shahabi	USA	59	PMB	LCNEC	Syn,NSE,CgA,CD56	7	IIIC	TAH,BSO,LND,optimal debulking	RT,irinotecan,cisplatin	DOD12
16	2019	Courtney Jenny	USA	56	PMB and pelvic pain	LCNEC + ECa	Syn,cytokeratin	16	IVB	TAH,BSO	Planned etoposide and cisplatin	DOD2
17	2010	Tadashi Terada	Japan	40	AUB	LCNEC + sarcomatous	Syn, cytokeratin, CD56,vimentin, CA125, CD34, ER, PR, p53, Ki-67, KIT, and PDGFRA	3	IB	TAH,BSO,omentectomy,LND	Unspecied chemo	AWD3
18	2008	Jorge Albores-Saavedra	Mexico	42	AUB	LCNEC	Syn,CgA,P16	4.5	IC	TAH	Platinum-based combination chemo	NED9
19	2011	Kedar K Deodhar	India	70	PMB and mild abdominal pain	LCNEC	Syn, CgA, CD56, cytokeratin,EMA,CD10, MIC-2, vimentin, smooth muscle actin	3.5	IVB	TAH,BSO,omentectomy	Etoposide, cisplatin	NED6
20	2013	My-Linh T. Nguyen	USA	71	PMB	LCNEC	Syn,CgA,CD56,p53,p16,PR	19.5	IVB	TAH,BSO,LND,omentectomy	Planned etoposide,cisplatin,and octreotide	DOD1
21	2004	Erhan	Turkey	52	PMB	LCNEC	Syn,NSE	NA	IC	TAH,BSO	Etoposide and cisplatin	DOD7
22	2012	Wyatt Unger	USA	62	PMB	LCNEC + ECa	Syn,CD56,p53,IMP-3	6	NA	TAH,BSO,LND,omentectomy	NA	NA
23	2014	Lan-xia Liu	China	87	PMB	LCNEC + ECa	Syn,CD56,CgA，CK7，AE1/AE3，EMA，p16，p53，EＲ，PＲ	10.5	IIIA	TAH,BSO	NA	NA
24	2014	Lan-xia Liu	China	44	AUB	LCNEC	Syn,NSE,CD56,CK7,p53	6	IIIC1	TAH,BSO,LND	NA	NA
25	2018	Yong-feng Ding	China	58	PMB	LCNEC + SCNEC + ECa	Syn,NSE,CgA,CD56,CD10，CK，EMA	0.9	IIIC	TAH,BSO,LND	Paclitaxel, carboplatin	NED21
26	2016	Cady E. Pocrnich	USA	54	NA	LCNEC	CgA	0.8	IA	TAH,BSO	RT	NED96
27	2016	Cady E. Pocrnich	USA	65	Bleeding	LCNEC + SCNEC + ECa	Syn,CgA,CD56,PanCK,CK18,p16,Pax-8,MLH1,PMS2,MSH2,MSH6	4.5	IA	TAH,BSO,LND	RT	DOD9
28	2016	Cady E. Pocrnich	USA	84	Bleeding	LCNEC + ECa	CD56,PanCK,CK18,p16,Pax-8,MSH2,MSH6	2.8	IB	TAH,BSO,LND	RT	NED118
29	2016	Cady E. Pocrnich	USA	66	Bleeding	LCNEC + ECa	Syn,PanCK,CK18,p16,MSH6	7.5	IB	TAH,BSO,LND	RT+Unspecied chemo	NED37
30	2016	Cady E. Pocrnich	USA	55	Bleeding	LCNEC + ECa	Syn,CgA	4.5	IB	TAH,BSO	NA	NA
31	2016	Cady E. Pocrnich	USA	47	Bleeding	LCNEC + ECa	Syn,CgA,PanCK,CK18,TTF-1, MLH1, PMS2,MSH2,MSH6	6	II	TAH,BSO,LND	RT+Unspecied chemo	DOD15
32	2016	Cady E. Pocrnich	USA	51	Bleeding	LCNEC + ECa	Syn,CgA,PanCK,CK18,p16,CD117,MLH1,PMS2,MSH2,MSH6	7	II	TAH,BSO,LND,omental biopsy	RT+Unspecied chemo	AWD11
33	2016	Cady E. Pocrnich	USA	68	Bleeding	LCNEC + SCNEC + ECa	Syn,CgA,CD56,p16,MSH2,MSH6	6	IIIA	TAH,BSO	RT+Unspecied chemo	NED24
34	2016	Cady E. Pocrnich	USA	69	Bleeding	LCNEC	CgA	NA	IIIA	TAH,BSO,LND	RT+Unspecied chemo	NED5
35	2016	Cady E. Pocrnich	USA	54	Bleeding	LCNEC + ECa	Syn,CgA,PanCK,CK18,p16,CD117,MLH1,PMS2,MSH2,MSH6	2.7	IIIB	TAH,BSO,LND	RT+Unspecied chemo	NED134
36	2016	Cady E. Pocrnich	USA	68	Bleeding	LCNEC + SCNEC + ECa	Syn,PanCK,CK18,p16,Pax-8, MLH1, PMS2,MSH2,MSH6	7.5	IIIB	TAH,BSO,LND,appendectomy	RT+Unspecied chemo	DOD13
37	2016	Cady E. Pocrnich	USA	52	Bleeding	LCNEC	CgA,PanCK,CK18,p16,CD117,MLH1,PMS2,MSH2	5.5	IIIC1	TAH,BSO,LND	RT+Unspecied chemo	NED66
38	2016	Cady E. Pocrnich	USA	55	Bleeding	LCNEC	Syn,CD56,PanCK,CK18,p16,MLH1,PMS2	Entire uterus	IIIC2	TAH,BSO,LND	None	DOD6
39	2016	Cady E. Pocrnich	USA	63	NA	LCNEC	Syn,CgA,CD56,PanCK,CK18,p16,Pax-8,MSH2,MSH6	9	IIIC2	TAH,BSO,LND,omental biopsy	NA	NA
40	2016	Cady E. Pocrnich	USA	87	Bleeding	LCNEC + SCNEC + ECa	Syn	4	IVB	TAH,BSO,omental biopsy	Unspecied chemo	DOD21
41	2016	Cady E. Pocrnich	USA	59	Dizziness(brain metastasis)	LCNEC + ECa	Syn,CgA,PanCK,CK18,p16,MLH1,PMS2,MSH2,MSH6	NA	IVB	TAH,BSO,LND	RT+Unspecied chemo	DOD12
42	2016	Cady E. Pocrnich	USA	55	Bleeding,abdominal pain	LCNEC + SCNEC + ECa	Syn,CgA,CD56,PanCK,CK18,p16,MLH1,PMS2,MSH2,MSH6	Entire uterus	IVB	TAH,BSO,appendectomy and soft tissue biopsy	NA	DOD3
43	2016	Cady E. Pocrnich	USA	37	Bleeding	LCNEC + SCNEC	Syn,CgA	6.2	IVB	TAH,BSO,omental and peritoneal biopsies	Unspecied chemo	DOD2
44	2016	Cady E. Pocrnich	USA	80	Dyspnea(lung metastasis)	LCNEC	Syn,CgA,PanCK,CK18,p16,CD117,MLH1,PMS2,MSH2,MSH6	NA	IVB	TAH,BSO, peritoneal biopsies	None	DOD3
45	2016	Cady E. Pocrnich	USA	55	Bleeding	LCNEC	Syn,PanCK,CK18,p16,Pax-8,MSH2,MSH6	12	IVB	TAH,BSO,LND,omental biopsies	Unspecied chemo	DOD9
46	2018	Lucinda Calheiros Guimarãesa	Brazil	75	Bleeding	LCNEC + ECa + melanocytic differentiation	Syn, CgA, cytokeratin (AE1/AE3) P16, CD56, and Melan-A	7.5	IIIA	TAH,BSO	RT+ Cisplatin and cyclophosphamide chemo	NED8
47	2019	Ruijiao Hu	China	54	AUB	LCNEC + SCNEC + serous carcinoma	Syn,CgA,P16, P53 , CK , Villin, and Ki-67	3	IIIC2	TAH,BSO,LND	Cisplatin and etoposide chemo	NA
48	2020	Liesel Elisabeth Hardy	Australia	47	Abdominal pain, distension, decreased appetite and loss of weight	LCNEC + high-grade serous adenocarcinoma	Syn,CgA,cytokeratins MNF116, CAM 5.2, EMAPAX8, ER, PR, CD99 and p16	13	IVB	Modified posterior exenteration, partial posterior vaginectomy, omentectomy and Hartmanns procedure with suboptimal debulking	RT,cisplatin and paclitaxel chemo,tamoxifen	NED92
49	2020	Glorimar Rivera	China	48	Increasing abdominal pain and girth	LCNEC + low-grade endometrial stromal sarcoma	Syn,CKs, chromogranin, PAX8, and Ki-67	7	NA	TAH,BSO	Cisplatin and etoposide chemo	DOD12
50	2021	Kotaro Inoue	Japan	65	Bleeding	LCNEC + SCNEC + ECa	Syn,CgA,CD56	5.5	IIIB	TAH,BSO,LND,omentectomy	Cisplatin and irinotecan chemo	NED3
51	2021	Ran Du	China	73	Bleeding	LCNEC	Syn, CgA,cytokeratin (AE1/AE3), CD56, P16, and Ki-67	5.1	IIIC	TAH,BSO	Paclitaxel liposome and lobaplatin chemo	NED15
52	2021	Utku Akgor	Turkey	70	Bleeding	LCNEC	Syn, CgA, CD56, and Ki-67	6.5	IVB	TAH,BSO,LND,omentectomy	None	DOD2
53	2023	Wing Yu Sharon Siu	China	55	Bleeding	LCNEC + ECa	CD56 ,vimentin, p53 ,ki67 (90%), and PMS2 (-)	8.5	II	TAH,BSO,LND,omentectomy	Cisplatin and etoposide chemo	NED5
54	2020	Present case	China	48	Bleeding	LCNEC	Syn,vimentin,desmin,Ki67	9	IIIC	TAH,BSO,LND	RT+irinotecan and cisplatin chemo	DOD19

1. AUB:abnormal uterine bleeding, 2. PMB:post menopausal bleeding, 3. LCNEC:large cell neuroendocrine cancer, 4. SCNEC:small cell neuroendocrine cancer, 5. ECa: endometrial cancer, 6. IHC: immunohistochemical, 7. Syn:synaptophysin, 8. NSE: neural-specific enolase, 9. CgA:chromogranin A, 10. TAH:total abdominal hysterectomy, 11. BSO:bilateralsalpingo-oophorectomy, 12. LND:lymphnodedissection, 13. RT: radiothrephy, 14. DOD:dead of disease, 15. NED:no evidence of disease, 16. AWD:alive with disease, 17. NA: not available.

### 4.2 Clinical manifestations

Most endometrial NEC have been reported in postmenopausal women and occasionally reported in perimenopausal or reproductive-age women. The most common clinical symptoms are abnormal vaginal bleeding and abdominal pain. Cases of paraneoplastic syndromes such as membranous glomerulonephritis, retinopathy, and Cushing's syndrome have also been reported. Some patients show no obvious clinical symptoms on physical examination.

### 4.3 Diagnosis

Endometrial LCNEC has no specific and sensitive tumor markers, and ultrasound outcomes are not obvious. CT and MRI are significant for preoperative evaluation, albeit the imaging performance is not specific. The diagnosis of NEC must rely on the outcomes of histopathology and immunohistochemical staining. WHO proposes that LCNEC should include the following characteristics: (1) polygonal cells with abundant cytoplasm and obvious nucleoli; (2) a neuroendocrine growth pattern of the tumor; and (3) >10% of the tumor cells expressed one or more neuroendocrine markers, such as CgA, Syn, and CD56 (Pocrnich et al., 2016).

Endometrial LCNEC can exist alone or occur with other tumors. Jenny et al. (2019) reviewed 20 cases of endometrial LCNECs, of which 8 were accompanied with endometrioid adenocarcinoma. Of these 8 cases, 1 also presented with serous components, 1 with squamous differentiation, 1 with sarcoma, and 1 with poorly differentiated adenocarcinoma. Guimarãesa reported an endometrial LCNEC with foci of melanocytic differentiation (Guimarães et al., 2018). Siu reported an endometrial LCNEC concomitant with Lynch syndrome (Siu et al., 2023). In addition, Albores-Saavedra (Albores-Saavedra et al., 2008) reported a case of endometrial LCNEC with partial sarcomatosis. Several cases of mixed growth of large- and small-cell NEC have been reported worldwide (Pocrnich et al., 2016, Inoue et al., 2021).

This present patient was a perimenopausal woman, with abnormal vaginal bleeding as the main symptom. Ultrasound and CT outcomes strongly indicated endometrial cancer. The final immunohistochemistry result was Syn (+), which supported the diagnosis of LCNEC.

### 4.4 Treatment

Presently, endometrial LCNEC does not have a unified treatment plan. Courtney (Jenny et al., 2019) reviewed 20 patients and found that, except for 2 patients who did not undergo surgery and 1 patient with only the uterus removed, the remaining opted for surgical resection of the whole uterus and double attachment. Of them 10 underwent with lymph node dissection, 6 underwent omentum resection, 7 underwent etoposide and platinum chemotherapy, 3 underwent irinotecan and cisplatin therapy, 2 underwent paclitaxel and carboplatin therapy, and 5 underwent adjuvant radiotherapy after the surgery. Pocrnich et al. (2016) analyzed 20 cases of endometrial LCNEC patients treated at the University of Texas Anderson Cancer Center from 1994 to 2014. All patients underwent surgical resection of the whole uterus and bilateral salpingo-oophorectomy, 13 underwent lymph node dissection, 5 underwent large omentum biopsy, 2 underwent appendectomy, 9 underwent adjuvant radiotherapy and chemotherapy, 3 received radiotherapy alone, and another 3 received chemotherapy only. Neoadjuvant chemotherapy was found to be beneficial against cervical small cell neuroendocrine cancer (Lee et al., 2008), although there exists no literature report for endometrial LCNEC. The patient in this case report was a perimenopausal woman diagnosed with endometrial LCNEC before surgery. Therefore, she underwent hysterectomy, salpingectomy, oophorectomy, pelvic lymph nodes, and para-aortic lymph node dissection. She received radiotherapy and cisplatin chemotherapy after the surgery.

### 4.5 Prognosis

Endometrial LCNEC has a poor prognosis, and early lymphatic or blood-way metastasis may occur (Dongol et al., 2014). Of the 54 patients, 38 had been diagnosed at least at stage III. The current patients are diagnosed at stage IIIC. Even early patients relapsed quickly after the treatment. The 5-year survival rate of high-grade neuroendocrine cancer was 14–39% (Höhn et al., 2021), and the prognosis was related to the tumor diameter (4 cm), FIGO stage, vascular invasion, parauterine involvement, depth of invasion, and other tumor types (Peng et al., 2012). Albores-Saavedra (Albores-Saavedra et al., 2008) believed that the stage and polypoid characteristics of the disease were the best indicators for predicting the disease. The disease-free survival time of all patients was 9 months to 7 years (average 47 months).

In summary, endometrial LCNEC is a highly malignant tumor that mostly affects postmenopausal women and occasionally affects perimenopausal or reproductive-age women. The most common clinical symptom is abnormal vaginal bleeding with giant polypoid mass often accompanied by deep infiltration of the uterine muscle wall. Histologically, it can be simple LCNEC, mixed NEC of the large and small cells, or with accompanying endometrioid adenocarcinoma, sarcoma, or serous carcinoma. The tumor cells expressed one or more neuroendocrine markers such as CgA, Syn, and CD56. Presently, there exists no unified treatment plan. Most of the cases were in the advanced stage at the time of detection, often with poor prognosis.

## 5 Ethics approval and consent to participate

Written informed consent was obtained from the patients and family meals, and the study was approved by Self-financing project of Guangxi Zhuang Autonomous Region Health and Family Planning Commission (Z2016288).

## Author contributions

Feng Yang collected case information and follow up, all authors contributed to the writing and editing of this study.

## CRediT authorship contribution statement

**Feng Yang:** Writing – original draft, Formal analysis. **Shoujun Liang:** Data curation. **Chuanzhong Liu:** Investigation. **Yeping Wei:** Methodology. **Liying Zhang:** Writing – review & editing, Conceptualization.

## Declaration of competing interest

The authors declare that they have no known competing financial interests or personal relationships that could have appeared to influence the work reported in this paper.
